# Combination of reduced post‐transplant cyclophosphamide and early tacrolimus initiation increases the incidence of chronic graft‐versus‐host disease in human leukocyte antigen‐haploidentical peripheral blood stem‐cell transplantation

**DOI:** 10.1002/jha2.962

**Published:** 2024-06-19

**Authors:** Toshiki Terao, Takumi Kondo, Makoto Nakamura, Hiroki Takasuka, Hideaki Fujiwara, Noboru Asada, Daisuke Ennishi, Hisakazu Nishimori, Keiko Fujii, Nobuharu Fujii, Yoshinobu Maeda, Ken‐ichi Matsuoka

**Affiliations:** ^1^ Department of Hematology and Oncology Okayama University Hospital Okayama Okayama Japan; ^2^ Division of Clinical Laboratory Okayama University Hospital Okayama Okayama Japan; ^3^ Division of Blood Transfusion Okayama University Hospital Okayama Okayama Japan

**Keywords:** chronic GVHD, haploidentical, hematopoietic stem‐cell transplantation, PTCy, tacrolimus

## Abstract

We evaluated the clinical impacts of the concurrent modification of post‐transplant cyclophosphamide (PTCy) dose and tacrolimus (Tac)‐initiation timing in 61 patients with human leukocyte antigen‐haploidentical transplantation. Reduced‐dose PTCy (80 mg/kg) was associated with a higher incidence of moderate‐to‐severe chronic graft‐versus‐host disease (GVHD) than standard‐dose PTCy (100 mg/kg) (35.0% vs. 26.6%, *p* = 0.053). Notably, early‐initiation Tac (day ‐1) increased moderate‐to‐severe chronic GVHD than standard‐initiation Tac (day 5) in the reduced‐dose PTCy group (*p* = 0.032), whereas Tac‐initiation timing did not impact chronic GVHD in the standard‐dose PTCy group. These data indicate that the combination of reduced‐dose PTCy and early‐initiation Tac can amplify chronic GVHD.

## 1 INTRODUCTION

Allogenic hematopoietic stem‐cell transplantation (HSCT) is the curative therapy for patients with hematological disorders. Recent data have shown that human leukocyte antigen (HLA)‐haploidentical transplantation with post‐transplant cyclophosphamide (PTCy)‐based graft‐versus‐host disease (GVHD) prophylaxis can be comparable overall survival (OS) or better GVHD‐free relapse‐free survival (GRFS) compared to conventional HSCT [1]. The previous study demonstrated that PTCy reduced the incidence of chronic GVHD as well as acute GVHD [1]. Although the PTCy regimen involves the administration of cyclophosphamide 50 mg/kg/day, day +3 and +4 in addition to the calcineurin inhibitors (CNIs) and mycophenolate mofetil (MMF) from day +5 [2], the modification of the PTCy regimen is a major challenge to expand the donor selection and improve the outcomes. Although previous studies have shown the feasibility of reducing the dose of PTCy (40 mg/kg/day) [3, 4], the concurrent modification of the PTCy dose and the initiation timing of CNIs is not well studied. In the previous studies, the early initiation of CNI showed better GRFS [5] or lower cytokine release syndrome (CRS) [6] compared to the standard initiation of CNI in combination with the standard‐dose PTCy (50 mg/kg/day). This is an encouraging result, however, theoretically, the early initiation of CNI impairs the effect of PTCy, that is, the elimination and the inactivation of alloreactive T‐cells expanding between transplantation and PTCy administration. In the murine model, the early initiation of CNI could inhibit donor T‐cell exhaustion and cause chronic GVHD [7]. Thus, the research on concurrent modification of PTCy dosage and the timing of CNI initiation is warranted.

We here evaluated the clinical outcomes of patients who received reduced PTCy and early initiation of tacrolimus (Tac) compared to those who received a standard PTCy regimen.

## 2 METHODS

We retrospectively reviewed 61 consecutive patients who underwent allogeneic peripheral blood HSCT with PTCy, Tac, and MMF for GVHD prophylaxis at our center between January 1, 2016, and June 30, 2022. The modification of PTCy (50 or 40 mg/kg/day, days +3 and +4) and Tac (initiating from days −1 or day +5) were mainly decided by the treating physicians based on the following principles. Briefly, patients who were older or had other comorbidities received a single immunosuppressant‐modified PTCy regimen, that is, reduced‐dose PTCy (40 mg/kg/day) or early‐initiation Tac (day −1). Patients with a high risk of relapse received the two immunosuppressants‐modified PTCy regimen, the reduced‐dose PTCy, and the early‐initiation tac. Otherwise, the patients received the standard PTCy regimen. All patients received MMF from day +5 to +30 at a dose of 15 mg/kg orally twice daily, with a maximum daily dose of 2000 mg. Other detailed methods are described in the Supporting Information Appendix.

## 3 RESULTS

**FIGURE 1:**
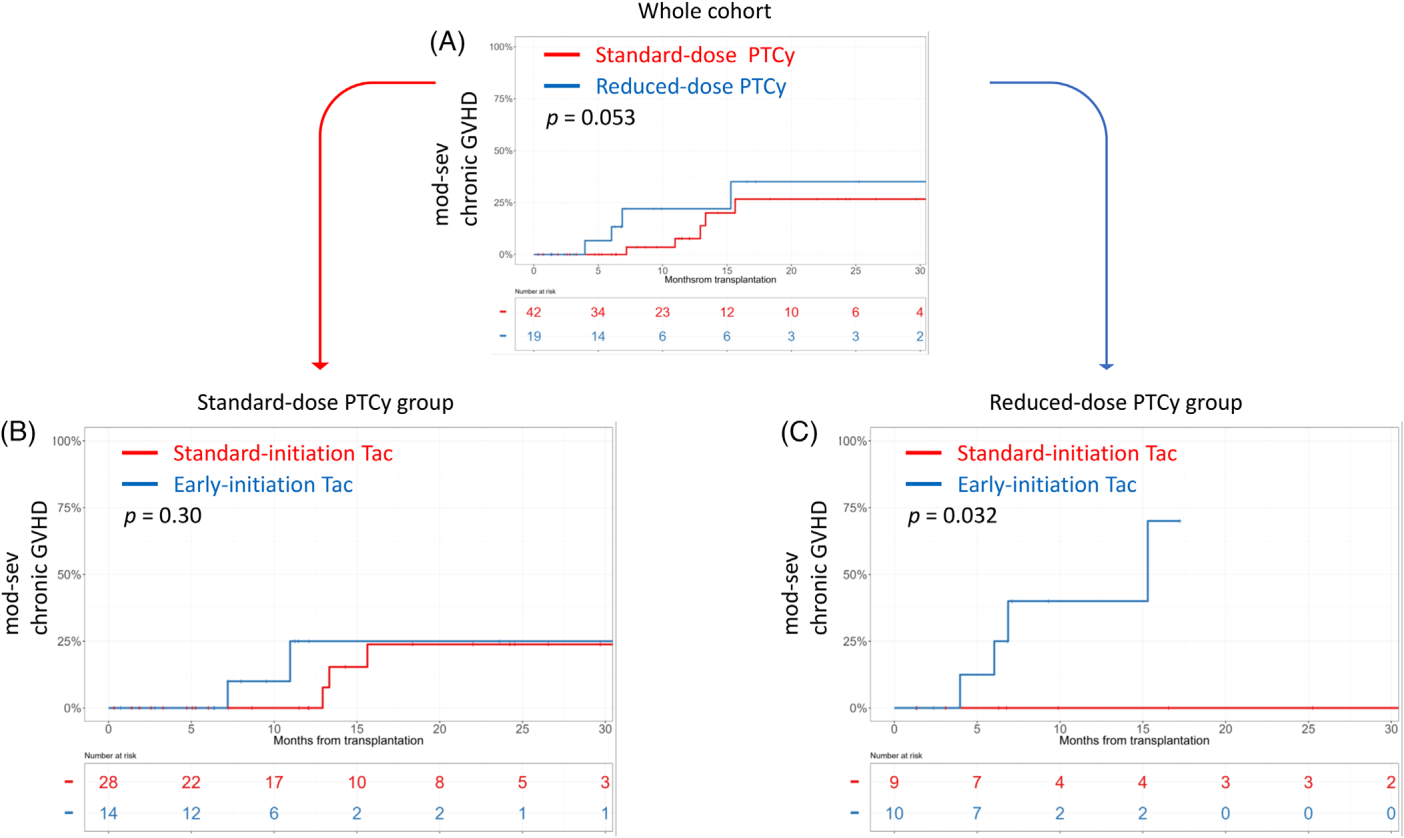
(A) Patients with reduced‐dose post‐transplant cyclophosphamide (PTCy) regimen showed higher moderate‐to‐severe chronic graft‐versus‐host disease (GVHD) at 2 years compared to those with standard‐dose PTCy regimen (35.0% vs. 26.6%, 95% confidence interval [CI] 13.8%–71.3% vs. 11.6%–54.0%, *p* = 0.053). (B) In the standard‐dose PTCy group, patients with early‐initiation tacrolimus (Tac) showed a similar incidence of moderate‐to‐severe chronic GVHD at 2‐years than those with standard‐initiation Tac (25.0% vs. 23.8%, 95% CI 6.6%–70.2% vs. 8.3%–57.3%, *p* = 0.30). (C) In the reduced‐dose PTCy group, patients with early‐initiation Tac showed a significantly higher incidence of moderate‐to‐severe chronic GVHD at 2 years than those with standard‐initiation Tac (NA vs. 0%, 95% CI NA–NA vs. NA–NA, *p* = 0.032).

First, we divided the whole cohort into two groups based on the PTCy dosage (Table 1) and evaluated the neutrophil and platelet engraftment, OS, the cumulative incidence of relapse (CIR), non‐relapse mortality (NRM), grade II–IV acute GVHD, and moderate‐to‐severe chronic GVHD. Compared to the patients with standard‐dose PTCy, those with reduced‐dose PTCy had significantly higher myeloid malignancy (89.5% vs. 50.0%, *p* = 0.004), worse performance status (Eastern Cooperative Oncology Group‐performance status ≥ 2, 36.8% vs. 4.8%, *p* = 0.003), and higher 2nd or 3rd transplantation (42.1% vs. 14.3%, *p* = 0.024). However, despite a lower CD34^+^ infused cells in the reduced‐dose PTCy group (4.4 vs. 5.0 × 10^6^cells/kg, *p* = 0.48), the median neutrophil engraftment was significantly earlier in the reduced‐dose PTCy group compared to the standard‐dose PTCy group (15 vs. 17 days, 95% confidence interval [CI] 15–17 vs. 14–17 days, *p* = 0.039, Figure S1A). Platelet engraftment was similar between both groups (Figure S1B). In terms of survival outcomes, the 1‐year OS and CIR were similar (Figure S1C,D), but the 1‐year NRM was almost significantly lower in the reduced‐dose PTCy group compared to the standard‐dose PTCy group (0% vs. 17.8%, 95% CI NA–NA vs. 8.9%–33.9%, *p* = 0.062, Figure S1E). The grade II‐IV acute GVHD was also similar between the two groups (Figure S1F), but the 2‐year moderate‐to‐severe chronic GVHD was almost significantly higher in the reduced‐dose PTCy group compared to the standard‐dose PTCy group (35.0% vs. 26.6%, 95% CI 13.8%–71.3% vs. 11.6%–54.0%, *p* = 0.053, Figure 1A).

**TABLE 1:** Patient characteristics.

	All	Standard‐dose PTCy (50 mg/kg/day)	Reduced‐dose PTCy (40 mg/kg/day)	
Factors	***N* = 61**	***n* = 42**	***n* = 19**	***p***
Age at transplantation, median (range)	52 (18–71)	50.5 (18–71)	52 (22–66)	0.55
Sex, male, *n* (%)	39 (63.9)	26 (61.9)	13 (68.4)	0.78
Female to male, *n* (%)	13 (21.3)	9 (21.4)	4 (21.1)	1
PS at transplantation, 2≥, *n* (%)	9 (14.8)	2 (4.8)	7 (36.8)	0.003
Diagnosis, *n* (%)^†^				0.004
myeloid malignancy	38 (62.3)	21 (50.0)	17 (89.5)	
non‐myeloid malignancy	23 (37.7)	21 (50.0)	2 (10.5)	
Disease status at transplantation				0.37
CR	42 (68.9)	27 (64.3)	15 (78.9)	
non‐CR	19 (31.1)	15 (35.7)	4 (21.1)	
Times of transplantation, *n* (%)				0.024
1st transplantation	47 (77.0)	36 (85.7)	11 (57.9)	
2nd or 3rd transplantation	14 (23.0)	6 (14.3)	8 (42.1)	
CMV serological status, *n* (%)				0.36
D+/R+	28 (47.5)	17 (42.5)	11 (57.9)	
D‐/R+	22 (37.3)	15 (37.5)	7 (36.8)	
Others	9 (15.3)	8 (20.0)	1 (5.3)	
DRI, high/very high, *n* (%)*	12 (25.5)	13 (32.5)	5 (26.3)	0.77
HCT‐CI, 2≥, *n* (%)**	28 (46.7)	18 (42.9)	10 (55.6)	0.41
Donor type, *n* (%)				0.94
sibling	18 (29.5)	13 (31.0)	5 (26.3)	
parent	6 (9.8)	4 (9.5)	2 (10.5)	
offsping	36 (59.0)	24 (57.1)	12 (63.2)	
unrelated	1 (1.6)	1 (2.4)	0 (0.0)	
Conditioning regimen, *n* (%)				1
MAC	29 (47.5)	20 (47.6)	9 (47.4)	
RIC	32 (52.5)	22 (52.4)	10 (52.6)	
Infused CD34+ cells, x10^6^/kg, median (range)	5.0 (2.2–11.5)	5.1 (2.4–10.4)	4.4 (2.2–11.5)	0.48
Total days of MMF, days, median (range)	37 (8–271)	37 (8–271)	37 (23–61)	
Total months of tacrolimus, months, median (range)	8.13 (0.3–62.0)	8.5 (0.3–50.0)	5.3 (1.3–62.0)	0.27

Abbreviations: CMV, cytomegalovirus; CR, complete remission; DRI, refined disease risk index; HCT‐CI, hematopoietic cell transplantation specific comorbidity index; MAC, myeloablative conditioning; MMF, mycophenolate mofetil; PS, performance status; PTCy, post‐transplant cyclophosphamide; RIC, reduced conditioning.

* *n* = 47.

** *n* = 60.

^†^ myeloid malignancy included AML, CML, MDS, and MPN. Nonmyeloid malignancy included ALL, lymphoma, and others.

We then divided each standard‐dose and reduced‐dose PTCy group into further subgroups based on the timing of Tac initiation, respectively, focusing on the onset of chronic GVHD. Although the timing of Tac administration did not impact the 2‐year moderate‐to‐severe chronic GVHD in the standard‐dose PTCy group (standard‐initiation Tac vs. early‐initiation Tac; 23.8% vs. 25.0% in the standard‐dose PTCy group, *p* = 0.30, Figure 1B), the early‐initiation Tac group showed significantly higher incidence of 2‐year moderate‐to‐severe chronic GVHD compared to the standard‐initiation Tac group in the reduced‐dose PTCy group (early‐initiation Tac vs. standard‐initiation Tac; NA vs. 0% in the reduced‐dose PTCy group, *p* = 0.032, Figure 1C). The severe chronic GVHD occurred in five patients (two in the reduced‐dose PTCy and early‐initiation tac group, the other three patients developed in each group, respectively) and the involvement organ was the lung. These severe chronic GVHD patients were alive without relapse until the end of the study period. On the other hand, the Tac initiation timing did not impact engraftment, OS, CIR, NRM, and grade II‐IV acute GVHD in both standard‐dose and reduced‐dose PTCy groups (Table S1).

## 4 DISCUSSION

In the present study, we evaluated the clinical impacts of the modification of PTCy‐based HSCT. Although the statistical differences in the incidence of moderate‐to‐severe chronic GVHD were not strikingly observed between the standard‐dose and reduced‐dose PTCy groups (*p* = 0.053), early‐initiation Tac significantly increased the incidence of moderate‐to‐severe chronic GVHD as compared to standard‐initiation Tac in patients receiving reduced‐dose PTCy (*p* = 0.032). However, the effect of Tac initiation timing on chronic GVHD was negated in patients receiving standard‐dose PTCy (Figure 1A–C). Our findings indicate that concurrent changes in CNI initiation and PTCy dosage could affect subsequent clinical outcomes, especially chronic GVHD.

The incidence of moderate‐to‐severe chronic GVHD at 2 years was 29.4%, which was similar to the previous reports (13–66%) [4, 8, 9]. However, in contrast to acute GVHD, the incidence of moderate‐to‐severe chronic GVHD clearly differed according to the modification of the dosage of PTCy and the timing of CNI initiation. The underlying mechanisms by which immunosuppressors of the PTCy method affect chronic GVHD would warrant further study. In a murine study, we recently reported that an initial increase in donor effector T‐cells in the bone marrow is related to the delay of immune reconstitution and the development of chronic GVHD but PTCy inhibits the alloreactive T‐cell response and restores intra‐bone marrow hematopoiesis [10]. As for the effect of CNIs on the PTCy regimen, an initial preclinical study showed that the early cyclosporine administration inhibited the proliferation of cyclophosphamide‐sensitive donor cells and interfered with tolerance induction [11]. Furthermore, a recent study also demonstrated that early cyclosporine administration inhibits donor T‐cell exhaustion and induces chronic GVHD [7]. Based on these basic data, a reduced‐dose PTCy and early‐initiation CNI could theoretically allow alloreactive T‐cell clones to survive without causing immune exhaustion, which potentially induces chronic GVHD. Therefore, the combination of reduced‐dose PTCy and early CNI administration should be carefully evaluated in terms of the incidence and severity of chronic GVHD, in addition to the risk of relapse and the patient's age or comorbidities, even if it may offer potential benefits through enhanced graft‐versus‐leukemia activity.

Our study has several limitations, including its retrospective nature, heterogeneous conditioning regimens, and diseases, and the lack of a validation cohort. In addition, our study included only a small number of patients, especially in the reduced‐dose PTCy group (*n* = 19), which makes it difficult to perform adequate statistical analysis. Long‐term follow‐up is warranted to evaluate the transplant outcomes in patients with reduced‐dose PTCy and early‐initiation Tac.

To conclude, despite the small size of the study cohort and short observation period, the strength of the study is that the incidence of moderate‐to‐severe chronic GVHD was clearly increased in the reduced‐dose PTCy and early‐initiation Tac group without increasing the risk of NRM. The clinical impact of the PTCy dosage and the timing of CNI administration should be reevaluated in a large sample, well‐designed, prospective study, taking into account the biological perspective.

## AUTHOR CONTRIBUTIONS

Toshiki Terao conceived and designed the study, collected data, performed the statistical analysis, wrote the manuscript, and provided patient care. Ken‐ichi Matsuoka designed and supervised the research and edited the paper. Takumi Kondo, Makoto Nakamura, and Hiroki Takasuka conducted the basic research related to the current clinical study. Hideaki Fujiwara, Noboru Asada, Daisuke Ennishi, Hisakazu Nishimori, Keiko Fujii, and Nobuharu Fujii collected data and provided patient care. Yoshinobu Maeda supervised this study and edited the paper. All authors reviewed and approved the manuscript.

## CONFLICT OF INTEREST STATEMENT

The authors have no competing interests.

## FUNDING INFORMATION

The authors did not receive financial support from any organization for the submitted work.

## ETHICS STATEMENT

All procedures performed in the study were in accordance with the ethical standards of the institutional and/or national research committee and the 1964 Helsinki Declaration and its later amendments or comparable ethical standards. The study was approved by the institutional review board (2209‐022).

## PATIENT CONSENT STATEMENT

This is a retrospective observational study and obtaining written consent was not mandatory. All participants did not provide any denial to this study documents that were made available to them in an opt‐out manner on the Web. The opt‐out method was approved by the institutional review board.

## CLINICAL TRIAL REGISTRATION

The authors have confirmed clinical trial registration is not needed for this submission.

## Supporting information

Supporting Information

Supporting Information

## Data Availability

The datasets generated during and/or analyzed during the current study are available from Toshiki Terao or Ken‐ich Matsuoka on reasonable request.
