# Supplementary figures and images for: Combination of reduced post‐transplant cyclophosphamide and early tacrolimus initiation increases the incidence of chronic graft‐versus‐host disease in human leukocyte antigen‐haploidentical peripheral blood stem‐cell transplantation
Source: EJHaem. 2024 Jun 19;5(4):810–4. doi: 10.1002/jha2.962 (PMC11327727; doi:10.1002/jha2.962)

Supplemental  
Figure 1

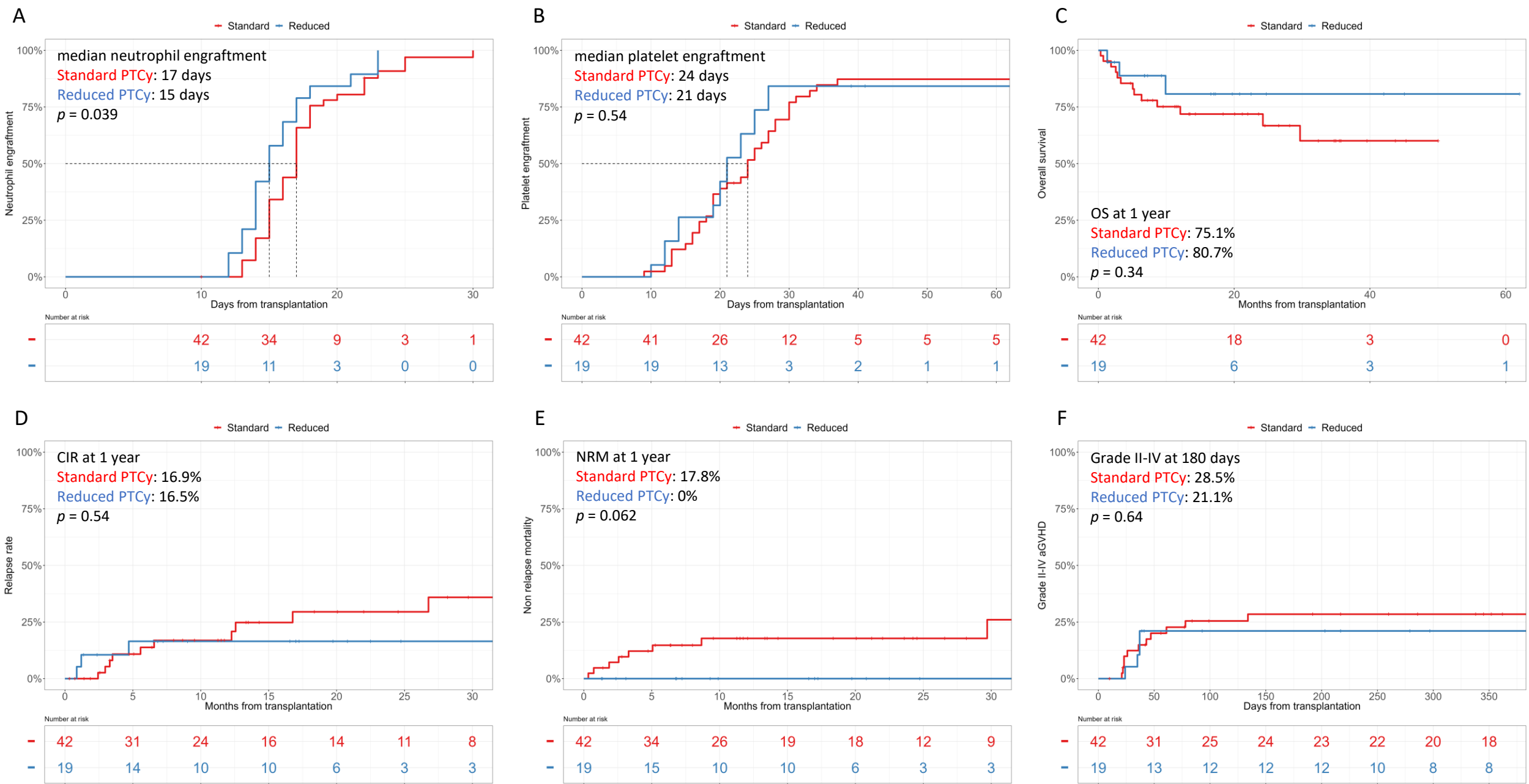

Supplement: Supplementary file 1 — Supporting Information [file JHA2-5-810-s002.pdf]
