# Supplementary material for: Combination of reduced post‐transplant cyclophosphamide and early tacrolimus initiation increases the incidence of chronic graft‐versus‐host disease in human leukocyte antigen‐haploidentical peripheral blood stem‐cell transplantation
Source: EJHaem. 2024 Jun 19;5(4):810–4. doi: 10.1002/jha2.962 (PMC11327727; doi:10.1002/jha2.962)
Supplement: Supplementary file 2 — Supporting Information [file JHA2-5-810-s001.docx]

**Supplemental File**

**Short Report**

**Title**

**Combination of reduced post-transplant cyclophosphamide and early tacrolimus initiation increases the incidence of chronic GVHD in HLA-haploidentical peripheral blood stem-cell transplantation**

Toshiki Terao^1^, Takumi Kondo^1^, Makoto Nakamura^1^, Hiroki Takasuka^1^, Hideaki Fujiwara^1^, Noboru Asada^1^, Daisuke Ennishi^1^, Hisakazu Nishimori^1^, Keiko Fujii^1,2^, Nobuharu Fujii^1,3^, Yoshinobu Maeda^1^, and Ken-ichi Matsuoka^1＊^

1 Department of Hematology and Oncology, Okayama University Hospital, 2-5-1, Shikata-cho, Kita-ku, Okayama, 700-8558, Japan.

2 Division of Clinical Laboratory, Okayama University Hospital, 2-5-1, Shikata-cho, Kita-ku, Okayama, 700-8558, Japan.

3 Division of Blood Transfusion, Okayama University Hospital, 2-5-1, Shikata-cho, Kita-ku, Okayama, 700-8558, Japan.

**Methods**

**Patient characteristics, conditioning regimen, and GVHD prophylaxis**

We retrospectively reviewed 61 consecutive patients who underwent allogeneic HSCT with PTCy, tacrolimus, and MMF for GVHD prophylaxis at our center between January 1, 2016, and June 30, 2022. G-CSF-mobilized peripheral blood stem cells (PBSCs) from related haploidentical donors were used as a graft source, with a target CD34^+^ dose of ≥ 4 × 10^6^/kg.

The conditioning regimen was determined by the physician. The MAC regimen was either fludarabine (Flu)150/intravenous busulfan (BU)12.8/total body irradiation (TBI)4Gy or Flu150/TBI12. The RIC regimen wase either Flu150/BU6.4/TBI4, Flu150/melphalan (Mel)80/TBI2, or FLU150/cyclophosphamide/TBI4.

**Definitions**

OS was defined as the time between HSCT and death. Relapse was defined based on morphologic evidence of ≥5% malignant cells in the bone marrow, other extra-medullary organs in myeloid malignancy, or progressive disease by the Lugano classification in malignant lymphoma ^1^. Non-relapse mortality (NRM) was defined as death without evidence of hematological disease relapse. Neutrophil engraftment was defined as the first day of three consecutive days with an absolute neutrophil count exceeding 0.5 × 10^9^/L after transplantation. Platelet engraftment was defined as the first day of seven consecutive days with an absolute platelet count exceeding 20 × 10^9^/L without platelet transfusion. Acute and chronic GVHD was diagnosed and graded based as previously published ^2,3^. Hematopoietic cell transplantation specific comorbidity index (HCT-CI) and refined disease risk index (DRI) were determined as described ^4,5^.

**Statistical analysis**

Baseline patient characteristics were compared using the Mann–Whitney U or Kruskal–Wallis test for continuous variables and the chi-squared or Fisher exact test for categorical variables.

The OS probability was estimated using the Kaplan–Meier method and compared using the log-rank test. The probabilities of neutrophil and platelet engraftment, acute and chronic GVHD, relapse, and NRM were estimated based on cumulative incidence methods and compared using Gray’s test. Competing events included death without neutrophil and platelet engraftment, death, or relapse without GVHD for acute and chronic GVHD, NRM for relapse, and relapse for NRM.

All statistical analyses were performed using R-software (version 3.6.1, The R Foundation for Statistical Computing, Vienna, Austria) in R-Studio. A two-sided *p* < 0.05 was considered statistically significant.

**Supplemental Figure Legends**

A: Patients with reduced-dose PTCy regimen showed an earlier neutrophil engraftment compared to those with standard-dose PTCy regimen (median 15 vs. 17 days, *p* = 0.039).

B: Patients with reduced-dose PTCy regimen showed a similar platelet engraftment compared to those with standard-dose PTCy regimen (median 21 vs. 24 days, *p* = 0.54).

C: Patients with reduced-dose PTCy regimen showed a similar OS at 1 year compared to those with standard-dose PTCy regimen (80.7% vs. 75.1%, 95% CI 50.4-93.5% vs. 58.6-85.8%, *p* = 0.34).

D: Patients with reduced-dose PTCy regimen showed a similar CIR at 1 year compared to those with standard-dose PTCy regimen (16.5% vs. 16.9%, 95% CI 5.6-43.0% vs. 7.9-33.9%, *p* = 0.54).

E: Patients with reduced-dose PTCy regimen showed a lower NRM at 1 year compared to those with standard-dose PTCy regimen (0% vs. 17.8%, 95% CI NA-NA vs. 8.9-33.9%, *p* = 0.062).

F: Patients with reduced-dose PTCy regimen showed a similar grade II-IV acute GVHD at 180 days compared to those with standard-dose PTCy regimen (21.1% vs. 28.5%, 95% CI 8.5-45.6% vs. 16.8-45.6%, *p* = 0.64).

| Supplemental Table 1  Transplant Outcomes | Standard-dose PTCy  (50 mg/kg, n = 42) |  |  | Reduced-dose PTCy  (40 mg/kg, n = 19) |  |  |  |
| --- | --- | --- | --- | --- | --- | --- | --- |
| Factors | Standard-initiation Tac  (day 5, n = 28) | Early-initiation Tac  (day-1, n = 14) | *p* | Standard-initiation Tac  (day 5, n = 9) | Early-initiation Tac  (day-1, n = 10) | *p* |  |
| Neutrophil engraftment  (median, 95% CI) | 17 (16-18) | 15 (14-17) | 0.14 | 16 (12-21) | 14.5 (12-16) | 0.43 |  |
| Platelet engraftment  (median, 95% CI) | 25 (19-30) | 21.5 (16-27) | 0.18 | 25 (10-27) | 20.5 (12-23) | 0.73 |  |
| OS (1-year, 95% CI) | 73.5% (52.2-86.4%) | 78.6% (47.2-92.5%) | 0.56 | 72.9% (27.6-92.5%) | 90.0% (47.3-98.5%) | 0.58 |  |
| CIR (1-year, 95% CI) | 13.3% (4.5-36.0%) | 23.1% (8.1-55.8%) | 0.44 | 23.8% (6.5-66.8%) | 10.0% (1.5-52.7%) | 0.52 |  |
| NRM (1-year, 95% CI) | 22.9% (10.9-44.1%) | 7.1% (1.0-40.9%) | 0.25 | 0% | 0% | NA |  |
| grade II-IV acute GVHD  (180-day, 95% CI) | 26.7% (13.7-48.1%) | 31.2% (12.9-63.6%) | 0.88 | 22.2% (6.1-63.5%) | 20.0% (5.4-59.1%) | 0.89 |  |
| **mod-sev chronic GVHD**  **(2-year, 95% CI)** | **23.8% (8.3-57.3%)** | **25.0% (6.6-70.2%)** | **0.3** | **0%** | **NA (NA-NA)** | **0.032** |  |
| Abbreviation: CI; confidence interval, CIR; cumulative incidence of relapse, GVHD; graft-versus-host disease, NRM; non-relapse mortality, OS; overall survival, PTCy; post-transplant cyclophosphamide | | | | | | |  |
|  |  |  |  |  |  |  |  |

**References**

1. Cheson BD, Fisher RI, Barrington SF, et al. Recommendations for initial evaluation, staging, and response assessment of Hodgkin and non-Hodgkin lymphoma: the Lugano classification. *J Clin Oncol*. 2014;32(27):3059-3068.

2. Przepiorka D, Weisdorf D, Martin P, et al. 1994 Consensus Conference on Acute GVHD Grading. *Bone Marrow Transplant*. 1995;15(6):825-828.

3. Filipovich AH, Weisdorf D, Pavletic S, et al. National Institutes of Health consensus development project on criteria for clinical trials in chronic graft-versus-host disease: I. Diagnosis and staging working group report. *Biol Blood Marrow Transplant*. 2005;11(12):945-956.

4. Sorror ML, Maris MB, Storb R, et al. Hematopoietic cell transplantation (HCT)-specific comorbidity index: a new tool for risk assessment before allogeneic HCT. *Blood*. 2005;106(8):2912-2919.

5. Armand P, Kim HT, Logan BR, et al. Validation and refinement of the Disease Risk Index for allogeneic stem cell transplantation. *Blood*. 2014;123(23):3664-3671.
